# Ring Augmentation and Pouch Resizing for the Treatment of Dumping Syndrome After Roux-en-Y Gastric Bypass: A Prospective Single-Center Trial

**DOI:** 10.1007/s11695-025-08245-1

**Published:** 2025-09-22

**Authors:** Jodok Matthias Fink, Mareike Luehn, Maximilian Meyer-Steenbuck, Gabriel Seifert, Sephan Herrmann, Stefan Fichtner-Feigl, Jochen Seufert, Goran Marjanovic, Katharina Laubner

**Affiliations:** 1 Centre for Surgery, Department of General and Visceral Surgery, Centre for Obesity and Metabolic Surgery, Medical Centre, University of Freiburg, Freiburg, Germany; 2 Division of Endocrinology and Diabetology, Department of Internal Medicine II, Medical Center - University of Freiburg, Faculty of Medicine, University of Freiburg, Freiburg, Germany

**Keywords:** Bariatric surgery, Banded gastric bypass, Ring augmentation, Dumping syndrome, Postbariatric surgery hypoglycemia

## Abstract

**Background:**

Early dumping syndrome (DS) and postbariatric surgery hypoglycemia (PBH) are common side effects after Roux-en-Y gastric bypass (RYGB) and may substantially impact patients’ quality of life. Established management options provide limited response in many patients. This trial tested the effect of pouch resizing and silicone ring implantation (trial intervention) in patients with dumping symptoms after RYGB.

**Methods:**

The trial intervention was assessed in a prospective single-arm trial including 16 patients. The primary endpoint was the change in Sigstad score 12 months after surgery. Secondary endpoints included Arts dumping score, insulin secretion, and glucose uptake assessed by a mixed meal tolerance test (MMTT), weight loss, quality of life, and safety evaluated by gastroscopy and contrast swallow.

**Results:**

The trial intervention led to a significant improvement in Sigstad (preOP: 20.4 (95% CI 17.8–23.1); 12 months: 10.4 (6.7–14.1); *P* = 0.0002) and Arts dumping score (preOP: 20.3 (16.7–23.9); 12 months: 10 (6.5–13.5); *P* = 0.0001). Glucose uptake and insulin secretion during MMTT remained largely unchanged (AUC glucose: 21,526 (19,654–23,398) mg/dl*min vs. 12 months 22,294 (20,511–24077) mg/dl*min), *P* = 0.21; AUC insulin: (preOP 62897 (33,531–92,263) pmol/l*min vs. 12 months 68,974 (40,691–97257) pmol/l*min, *P* = 0.19). Following the trial intervention, mean weight loss was 5.1% (1.4–8.9%). No ring migration or misplacement occurred during follow-up.

**Conclusions:**

Pouch resizing and silicone ring implantation led to a clinically relevant improvement in symptoms associated with DS and PBH. This was not related to an apparent impact on glycemic regulation.

**Graphical Abstract:**

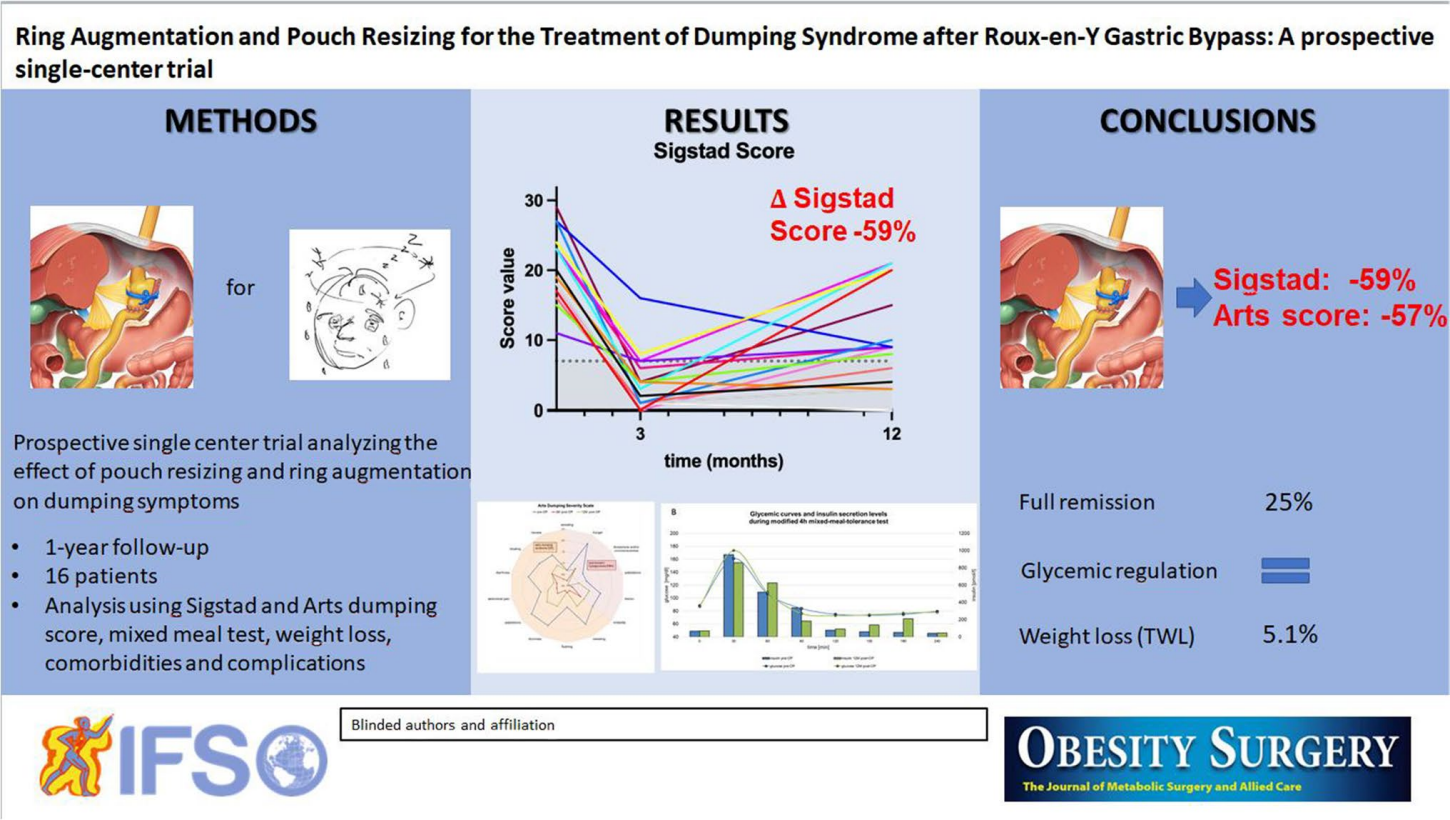

## Introduction

Dumping syndrome (DS) and postbariatric surgery hypoglycemia (PBH) occur in up to 40% after bariatric surgery [1, 2]. DS is triggered by osmotic pressure of nutrients and subsequent diffusion of fluid into the bowel lumen, while in PBH, consumed carbohydrates cause exaggerated insulin/GLP-1 release and subsequent hypoglycemia [2, 3]. Most patients can control their symptoms by consumption of small meals with a low carbohydrate content [4]. Approx. 2.5% of patients develop substantially impaired quality of life [4]. Unless bypass anatomy as precondition for DS and PBH is reversed, efficient treatment for these patients is challenging [1, 5, 6]. Treatment targets diminishing insulin response and slowing gastrointestinal transit [7]. Dietary advice, combining small meals with a low-glycemic index, high fiber, and protein, with slow eating and well chewing is the primary approach. If dietary intervention is not successful, the addition of pharmacotherapy may lead to better symptom control. Scientific data with positive yet variable results are available for acarbose, diazoxide, somatostatin analogues, calcium-channel blockers, sitagliptin, GLP-1-receptor agonists (GLP-1RA), and SGLT2-inhibitors [1, 3, 8]. Furthermore, continuous glucose monitoring (CGM) has been associated with reduced glycemic variability and allows for earlier intervention in patients with PBH [9]. Endoscopic pouch outlet reduction led to resolution of DS in 57% 2 years following the procedure [10]. A retrospective single-center series primarily analyzing revisionary surgery for weight regain after Roux-en-Y gastric bypass (RYGB) demonstrated a relief of dumping symptoms in 58% following silicone ring implantation [11].

Given limited efficiency of medical and surgical approaches and largely missing prospective scientific data, we tested if silicone ring implantation and pouch resizing lead to clinical symptom improvement of DS/PBH in a prospective single-arm trial.

## Materials and Methods

The study protocol was approved by the local ethics committee and conducted in accordance with the principles of the Declaration of Helsinki. All patients gave written informed consent.

### Study Design

The study is a single-center, open-label, single-arm trial including patients suffering from DS or PBH following RYGB (DRKS00020293).

### Participants

Patients $\ge$ 18 years with relevant dumping symptoms following RYGB, who had undergone a failed conservative approach for dumping treatment, and were indicated for pouch resizing plus silicone ring placement were eligible to participate in this study. Surgical revision was indicated after discussion in a multidisciplinary team based on insufficient response to dietary modifications, clinically relevant dumping symptoms impairing quality of life or everyday functioning and failed or declined pharmacological dumping treatment. Patients of this study initially received the RYGB operation between May 2013 and August 2019. The majority (10/16) of patients was primarily operated at the author’s institution. During this time, 651 patients had received a primary RYGB. The first patient was included in May 2020, the last patient on October 2022. In this period, 242 patients were treated for dumping by the multidisciplinary team, whereas 185 received medical therapy and 7 an endoscopic transoral outlet reduction (TORe).

Formal study inclusion criteria were defined as Sigstad score > 7 and abnormal values during the mixed meal tolerance test (MMTT; increase in heart rate by 10%, drop in systolic blood pressure by 10% within the first 30 min or serum glucose value < 60 mg/dl or 3.3 mmol/l). The combination of a MMTT with clinical scores was the best-established characterization for a dumping syndrome at that time [2]. Therefore, this combination was utilized not only for study inclusion but also for follow-up examinations despite the circumstance that a liquid glucose test may underestimate changes induced by pouch restriction.

Exclusion criteria were insulin-dependent type 2 diabetes (T2D), type 1 diabetes, pregnancy, untreated psychiatric illness, alcohol or drug abuse, liver cirrhosis, and Crohn’s disease.

### Intervention

Patients received a complete adhesiolysis of the pouch and gastrojejunostomy (GJ). Pouch dilatation was assessed by air insufflation. Pouch resizing was conducted alongside a 35 French bougie using 2–3 loads of a linear Endo-GIA device (Medtronic, purple cartridge). The ring (MiniMizer, Bariatric Solutions) was implanted in a perigastric technique, positioned 3–4 cm below the esophago-gastric junction and a minimum of 2 cm above the GJ. As described previously, constant pressure on the gastric wall should be avoided in a ring-augmented procedure [12]. Therefore, the ring was closed to leave a circumference of 7.5 cm. Including bougie size and stomach wall thickness, this enabled a loose ring placement.

### Outcome Measurements

Primary outcome was defined as Sigstad score after 12 months. The score value was determined at inclusion, 3 and 12 months after revisional surgery.

#### Secondary Outcomes

Clinical outcome measurements including Sigstad score and Arts dumping severity scale were determined at inclusion, 3 and 12 months postoperatively. MMTT, barium swallow, and gastroscopy were performed at 0 and 12 months.

Carbohydrate tolerance was recorded semi-quantitively as carbohydrate intake as (a) avoided completely, (b) partially possible, and (c) possible without limitation. Additional medical therapy of DS/PBH, number of antihypertensive agents, and nicotine use were recorded.

For the MMTT, blood samples were taken at the beginning and every 30 min up to 240 min following ingestion of 200 ml of Fresubin energy drink (Fresubin® energy fiber drink). Items recorded included insulin, glucose, hematocrit, heart rate, and blood pressure. During gastroscopy, the presence of hiatal hernia and reflux esophagitis, ring position, and pouch length were assessed. In addition, contrast reflux was assessed during barium swallow.

T2D was evaluated recording HbA1c and use of anti-diabetic medication. Quality of life was evaluated using the Bariatric Analysis and Reporting Outcome System (BAROS). Reflux symptoms and regurgitation were documented semi-quantitatively as either not present, incidence of $\ge$ 1/week, or $\ge$ 1/month. Additionally, a reflux questionnaire was used (Reflux Symptom Index (RSI)).

Postoperative complications were defined as early/late or major/minor by the guidelines for outcome reporting of the American Society for Metabolic and Bariatric Surgery [13].

### Statistical Analysis

Sample size was calculated to detect a difference in Sigstad score 12 months after revision compared to baseline applying a sign-rank test. It was assumed that ring implantation would lead to score improvement in 90% of patients. Based on a two-sided significance level of 0.05, 12 patients were required. Due to a high uncertainty based on limited data availability and to account for drop-outs, sample size was adjusted to 16 [14].

Continuous variables were characterized using mean and confidence interval (CI). Categorical variables were summarized using frequencies or percent of patients in each category. For MMTT evaluation, area under the curve was calculated for glucose and insulin. A Wilcoxon signed rank test was used to calculate median treatment differences of continuous variables.

To obtain dichotomous outcomes, reflux symptoms, regurgitation, and dysphagia were grouped as either not present or present at any degree.

All statistical tests were performed at the two-sided 0.05 significance level. Prism 8.3 for macOS (GraphPad Software, LLC) was used for statistical analysis. A *P*-value < 0.05 was considered significant.

## Results

**Fig. 1:**
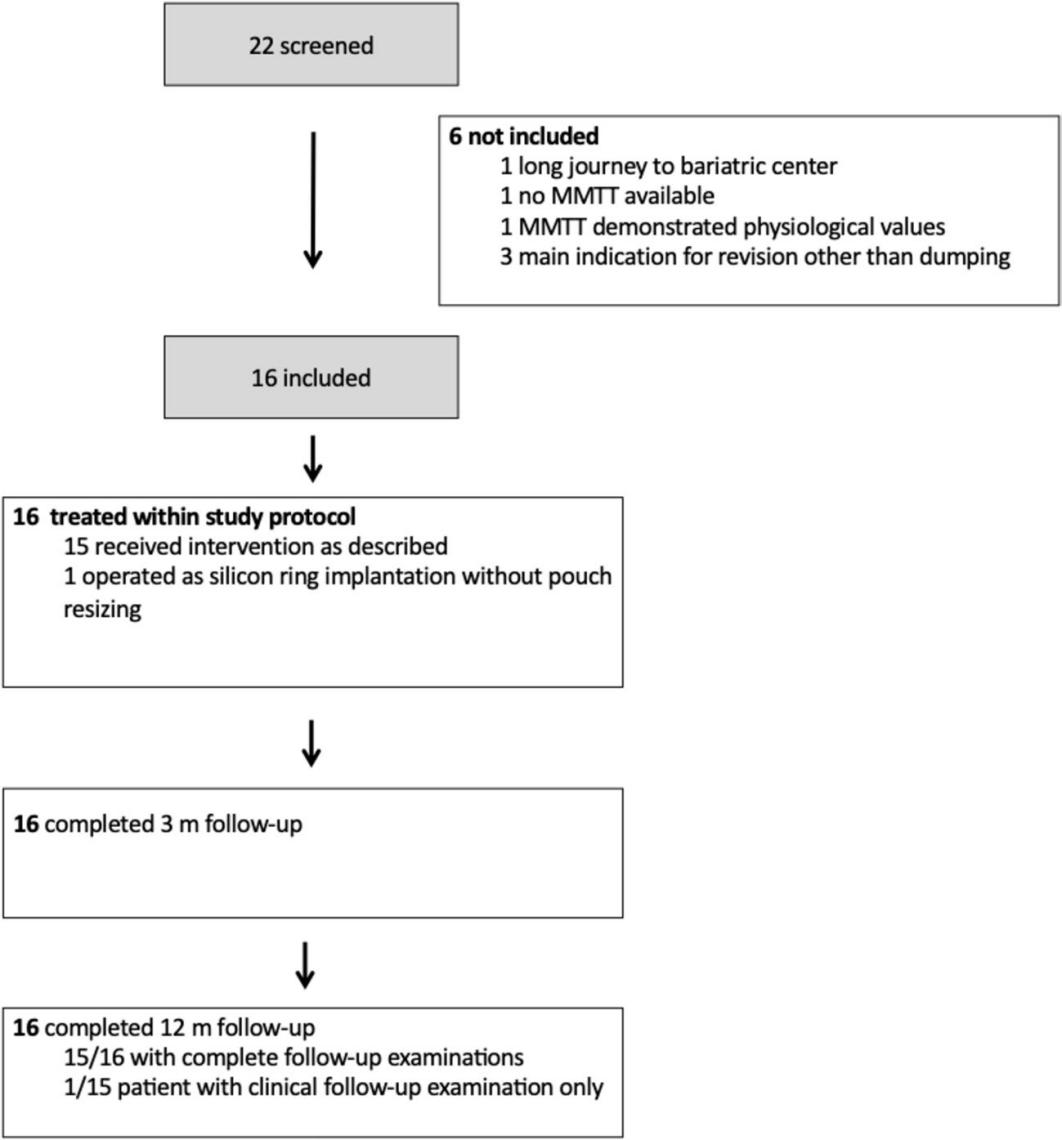
Patient disposition in trial analyzing the effect of silicone ring implantation and pouch resizing on dumping syndrome and postbariatric surgery hypoglycemia

Among the 16 patients recruited, RYGB was the primary bariatric operation in 15 patients. One patient had been previously converted from a sleeve gastrectomy to a RYGB due to severe reflux. Silicone ring implantation was conducted at a mean latency of 56 (42.4–69.7) months after RYGB. Gastric pouch was resized in 15 patients. One patient with a narrow pouch did not require resizing. Candy cane was resected in six patients; four patients received an additional hiatoplasty. Duration of surgery was 78.1 (66.5–89.6) min. Baseline patient characteristics are displayed in Table 1. Figure 1 depicts the flow of patients throughout the study.

**Table 1:** Baseline patient characteristics

Characteristic	
Age, mean (CI), years	46.0 (41.4–50.6)
Female, No. (%)	16 (100)
Weight, mean (CI), kg	96.8 (88.4–105.3)
BMI, mean (CI), kg/m^2^	35.7 (32.5–38.8)
Type 2 diabetes, *n* (%)	1 (6.25)
Arterial hypertension, *n* (%)	2 (12.5)
Gastroesophageal reflux complaint $\ge$ 1/week, *n* (%)	2 (12.5)
Refluxesophagitis, *n* (%)	1 (6.25)
RSI score, mean (CI)	5.8 (1.9–9.7)
PPI use, *n* (%)	10 (62.5)
Hiatal hernia, *n* (%)	6 (37.5)
Nicotine use, *n* (%)	4 (25)
Pouch length, mean (CI), cm	6.4 (5.7–7.1)

*BMI* body mass index, *RSI* reflux symptom index, *PPI* proton pump inhibitor

### Primary Endpoint

**Fig. 2:**
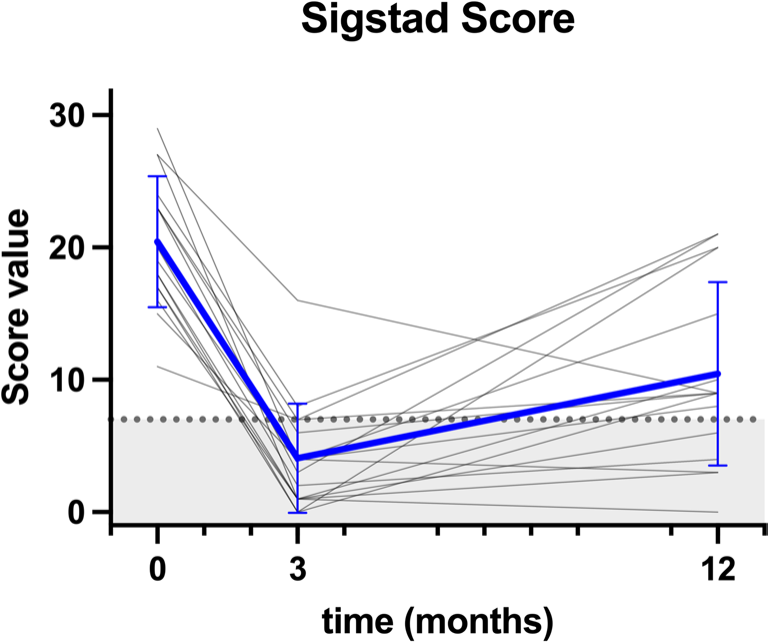
Spaghetti plot of Sigstad score values of each individual patient from baseline through 3 and 12 months post intervention as well as mean values (bold blue line, ± standard deviation). The grey are fill defines the range of normal Sigstad score values (score value 0–7)

Sigstad score decreased from 20.4 (17.8–23.1) to 10.4 (6.7–14.1; *P* = 0.0002). The median calculated score difference was −12 (−16 to −2). Sigstad score diminished by $\ge$ 50% in 88% of patients 3 months after revision and in 56% after 12 months. Only four patients presented with formally non-pathological Sigstad score values (score < 7, Fig. 2, Table 2). The largest reduction was seen in the score items “weakness” and “sleepiness” (both 94 to 44%), the lowest for the score item “palpitations” (81 to 63%). Hiatoplasty or candy cane resection had no influence on Sigstad score improvement.

**Table 2:** Individual Sigstad and global Arts score values

Patient	Time interval					
	Preoperatively		3 months		12 months	
	Sigstad	Arts	Sigstad	Arts	Sigstad	Arts
1	20	21	2	4	4	4
2	17	13	0	0	20	13
3	19	20	4	6	3	5
4	24	33	8	13	20	21
5	15	17	4	1	8	5
6	23	31	3	7	21	19
7	27	23	1	1	10	11
8	27	21	16	10	9	8
9	11	8	7	1	9	9
10	23	20	7	5	21	13
11	23	30	6	14	9	20
12	29	22	4	3	15	15
13	16	18	1	3	6	11
14	18	20	1	0	3	0
15	17	12	1	0	0	1
16	18	16	0	2	9	5

### Secondary Endpoints

#### Arts Dumping Severity Scale

**Fig. 3:**
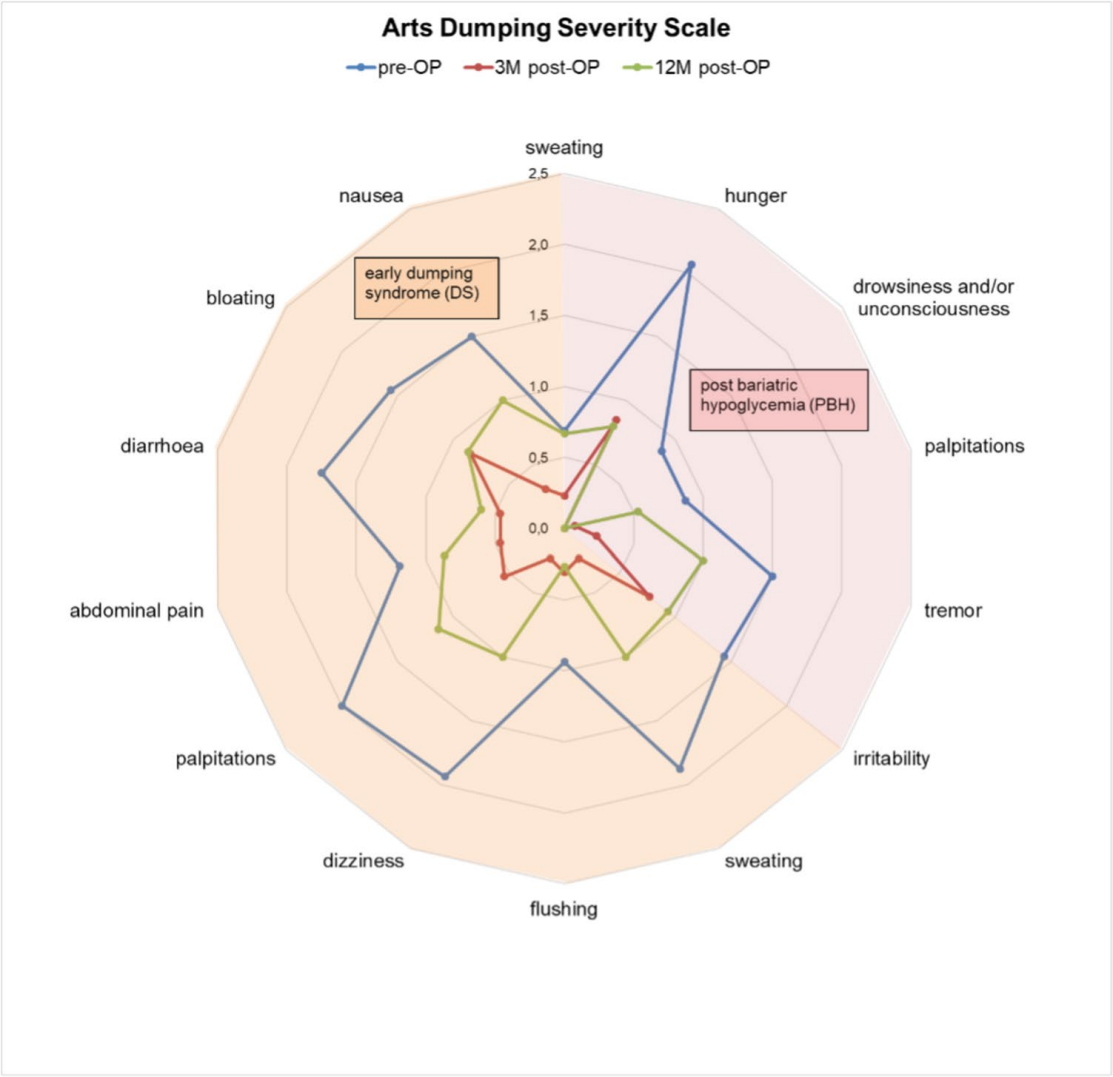
Mean score values of every item assessed in the Arts dumping severity score from baseline through 3 and 12 months post intervention. Score items related to (early) dumping syndrome (DS) are depicted in the left half of the spider plot, items related to PBH are recorded on the right

Arts dumping severity scale globally decreased by a median of 11.5 (−13 to −7; *P* = 0.0001, Table 2) points. Score value dropped from 20.3 (16.7–23.9) to 10 (6.5–13.5). Of note, trial intervention was equally effective to reduce score values for DS (12.9 (10.8–15.0) to 6.3 (3.9–8.7); *P* = 0.0001) and PBH (7.4 (CI 4.9–10) to 3.7 (1.7–5.6); *P* = 0.0018). Individual values are displayed in Fig. 3.

#### MMTT and Clinical Outcome Parameters

**Fig. 4:**
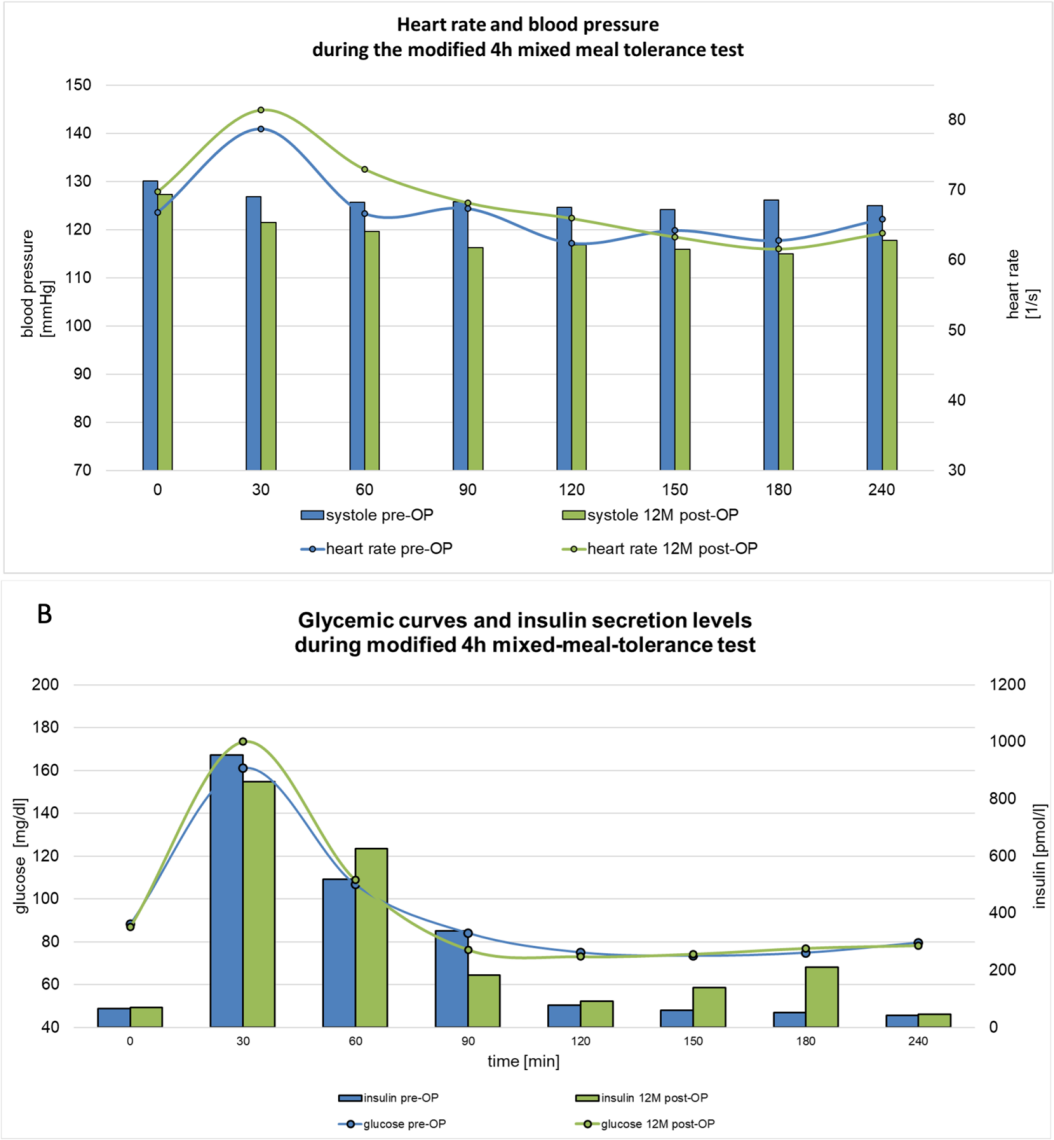
**A** Heart rate and systolic blood pressure values during the MMTT. Heart rate is presented as connected dots, blood pressure values as bars at each individual time point. Blue: preoperatively assessed values, green: 12-month follow-up. **B** Glucose and insulin values during the mixed meal tolerance test (MMTT). Glucose values are presented as connected dots, insulin values as bars at each individual time point. Blue: preoperatively assessed values, green: 12-month follow-up

MMTT was indicative for DS in 13 patients at trial entry, whereas the majority (11 of 13) reached the cut-off by an increased heart rate. The threshold for PBH was met by seven patients. Pathologic values in the MMTT normalized with respect to DS in four (31%) patients, regarding PBH in three (43%) patients. Heart rate increase declined from 18.4 b/min (13.9–22.9) to 13.1 b/min (6.8–19.3) after intervention (*P* = 0.14; Fig. 4 A). The MMTT performed 1 year after silicone ring implantation demonstrated largely unchanged AUC values for glucose (preOP 21526 (19,654–23,398) mg/dl*min vs. postOP 22294 (20,511–24,077) mg/dl*min; *P* = 0.21) and insulin (preOP 62897 (33,531–92,263) pmol/l*min vs. postOP 68974 (40,691–97257) pmol/l*min; *P* = 0.19; Fig. 4B). Carbohydrates were partially tolerated or avoided by seven (43.8%) patients preoperatively. After the study intervention, this proportion increased to 68.8%. Subset BAROS score value for quality of life increased from 0.6 (−0.3 to 1.5) before revision to 1.1 (0.3–2; *P* = 0.06).

#### Weight Loss

**Fig. 5:**
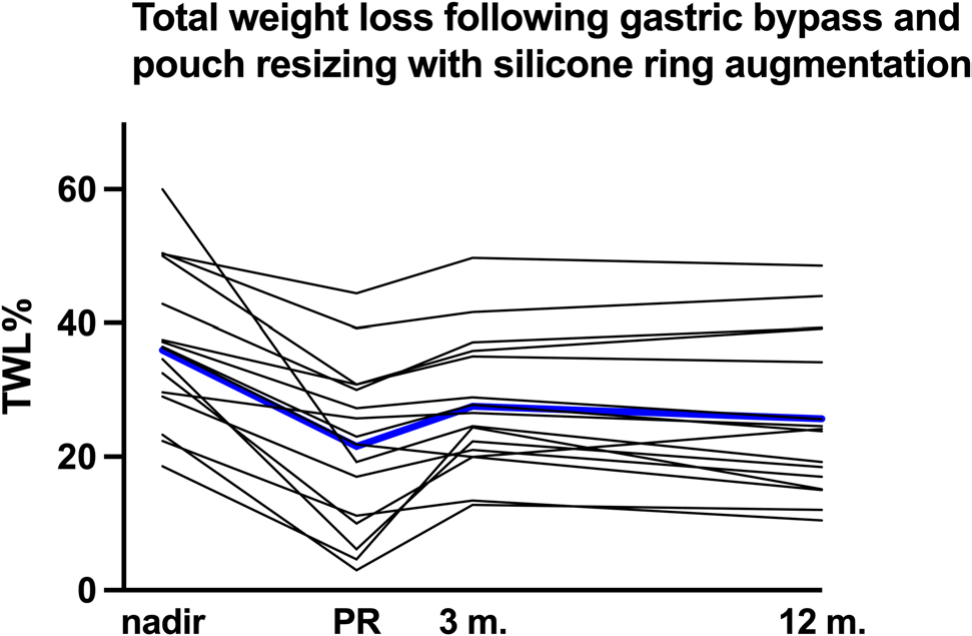
Total weight loss of each individual patient from nadir, time of pouch resizing, and silicone ring augmentation through 3 and 12 months post intervention. Mean total weight loss of all patients is depicted as blue (bold) line. TWL%, total weight loss; nadir, nadir weight loss; PR, pouch resizing and silicone ring implantation; m., months

Nadir total weight loss (TWL) after RYGB in this cohort was 36% (30.7–41.3; Fig. 5). Prior to silicone ring implantation, TWL had decreased to 21.5% (15–28.1). Eight patients had regained > 20% of body weight, four patients more than 30%. Excess weight loss was < 50% in eight patients. Twelve months following the study intervention, mean weight loss was 5.1% (1.4–8.9; *P* = 0.03) or 5.2 kg (1.4–9.0; *P* = 0.02). Six patients did not experience any weight loss; five patients lost > 10%. Weight loss after silicone ring implantation correlated with weight regained from nadir weight (Pearson *r* 0.55).

#### Safety and Comorbidities

Surgical complications Clavien-Dindo Class I occurred in two patients. One patient experienced a postoperative staple line bleeding which required operative revision (Clavien-Dindo IIIb). Refluxesophagitis (grade A) was detected in one patient before revision and persisted in the control gastroscopy. RSI values remained unchanged (initially 5.8 (1.9–9.7); trial end 5.5 (2–9), *P* = 0.88). The proportion of patients using PPIs dropped from 63 to 38%. During gastroscopy, the impression of the silicone ring was measured 3 cm (2.3–3.7) below the gastroesophageal junction. Ring migration or displacement could not be detected. Regurgitation was present in five (31.3%) patients preoperatively and after silicone ring implantation (three patients with persisting regurgitation and two patients with newly diagnosed symptoms).

## Discussion

This is the first prospective trial analyzing symptoms of DS and PBH following silicone ring augmentation. This intervention led to a sustained Sigstad score improvement by 50% or more at 12 months in 56% of patients. The improvement in Arts dumping score suggested a similar effect on symptoms of DS and PBH. Full dumping symptom resolution was achieved in 25% of patients. In contrast, 1-year retrospective data on silicone ring implantation without pouch resizing in 24 patients demonstrated a resolution of 58%. However, dumping symptoms were measured only semi-quantitatively in this trial [11]. For TORe, resolution of dumping was described in 67% at 1 year in a set of patients with a considerably lower initial Sigstad score [10]. This may have altered the rate of complete dumping resolution observed in these studies. Furthermore, TORe may not be ideal in patients with dilated gastric pouches, as outlet reduction in these patients potentially leads to regurgitation. Acarbose, considered to be first-line medical treatment in patients with PBH, led to lower glucose values in a MMTT performed in 11 patients after RYGB and documented PBH, demonstrating the principal effect of this medication. However, acarbose could not decrease the number of hypoglycemic episodes in an outpatient setting in the same trial [7]. Data from a multicenter case series including 22 patients with DS or PBH demonstrated at least a partial response in 3 of 6 patients for diazoxide and in 8 of 13 patients for a somatostatin analogue [15]. Lately, GLP-1RA are offered to patients with PBH targeted at reduction of gastrointestinal motility, yet promising data are still limited to a small body of studies [3]. Clearly, these studies revealing a success rate in often less than two out of three patients demonstrate the challenges of dumping therapy following RYGB. In this constellation, pouch resizing and ring augmentation may be positioned as alternatives to eTOR in second-line treatment after failed medical dumping therapy or for patients unwilling to take lifelong medication. When eTOR has a lower rate of severe complications such as staple line leakage, pouch resizing and silicone ring augmentation could be favorable in patients with dilated gastric pouches [10, 16].

Patients in this study reported a drastic reduction in almost every item of the Arts dumping score. Especially for characteristic DS items such as diarrhea, palpitations, and dizziness, or items related to PBH such as postprandial hunger, score rating went from “relevant” to “mild.” None of the patients reported drowsiness following the trial intervention. Despite these findings suggesting a retraceable pathophysiologic change, the standardized liquid MMTT revealed largely unchanged glycemic values and vital signs. In light of the aforementioned changes, we would have expected a slower rise in glucose values and vital signs, a later peak, and a smaller AUC as indirect evidence for delayed gastric emptying following silicone ring implantation and pouch resizing. Possibly, a liquid MMTT lacks the precision to detect this change as delayed gastric emptying after silicone ring implantation had previously been demonstrated by a primarily solid test meal [17].

On the other hand, it is plausible to assume that ring implantation led to consumption of smaller meals. This is a cardinal goal in dumping treatment and may explain the observed benefit. Meal size was not explicitly recorded in this trial.

A recently published meta-analysis on revisional treatments for impaired weight loss after RYGB reported a TWL of 17.2% after pouch resizing and 13.6% for silicone ring implantation [16]. Weight loss in the current trial was considerably lower. However, the indication for surgery in the current trial was focused on dumping, with only half of the patients having experienced insufficient weight loss or weight regain. Comparing good and poor responders, Boerboom identified patients with weight regain after previous good weight loss to benefit most from secondary silicone ring implantation [18]. The correlation of weight regained and weight lost after revision in the current trial identifies a similar patient subgroup for a good response after revision in this respect.

This study has several limitations. Diagnosis of dumping syndrome varies largely on the test used, and neither clinical nor functional tests have a high specificity for the diagnosis of DS or PBH [19]. For example, the MMTT cut-off used for PBH in the current trial was 3.3 mmol/l. Possibly, a stricter cut-off of 3 mmol/l or 2.8 mmol/l would have led to a more rigorous patient selection [2, 20]. Nonetheless, patients required pathologic findings in both MMTT and Sigstad score for study inclusion. The study design, with repeated measures in the same patient, limited inter-patient variability.

Functional testing in the current trial was confined to a MMTT with a chemically defined meal. Although considered standard of care, this may not be ideal to evaluate treatment effects in patients with DS or PBH [2]. Despite growing experience with GLP-1 receptor agonists as an effective treatment option for the aforementioned patient group, liraglutide had no effect on glucose or insulin response to a MMTT but led to a lower frequency of hypoglycemic episodes in continuous glucose monitoring of the same patients [7]. Patients of the current trial did not receive continuous glucose monitoring to assess functional results in a real-world setting. Although all patients had received dietary counselling prior to study inclusion, postoperative diet was not formally standardized. Nonetheless, carbohydrate consumption is relevant for the assessment of PBH. In relation to trial initiation, patients’ carbohydrate tolerance was decreased at 12-month follow-up. This, despite dietary counselling before revision, may be due to an increased awareness of symptom triggers throughout patient treatment in this trial. This may, in part, explain the patients’ clinical benefits independently of ring implantation. Furthermore, the study intervention was a combined intervention of pouch resizing and silicone ring implantation. This design does not allow to discriminate which part of the intervention contributed to the effects reported.

## Conclusion

Silicone ring implantation and pouch resizing offer an effective therapeutic strategy for symptom control in patients with DS and PBH independent of effects on glycemic dynamics. Complete resolution of dumping symptoms is only possible for a minority of patients.

## Data Availability

No datasets were generated or analysed during the current study.
